# Analysis of MicroRNA Expression in Newborns with Differential Birth Weight Using Newborn Screening Cards

**DOI:** 10.3390/ijms18122552

**Published:** 2017-11-28

**Authors:** Patricia Rodil-Garcia, Elvira del Carmen Arellanes-Licea, Angélica Montoya-Contreras, Luis A. Salazar-Olivo

**Affiliations:** Molecular Biology Division, Instituto Potosino de Investigación Científica y Tecnológica, Camino a la Presa San José 2055, San Luis Potosí 78216, SLP, México; patyrodil@gmail.com (P.R.-G.); elvira.arellanes@utcorregidora.edu.mx (E.d.C.A.-L.); angelica.montoya@ipicyt.edu.mx (A.M.-C.)

**Keywords:** circulating microRNAs, newborn screening cards, birth weight, fetal programming

## Abstract

Birth weight is an early predictor for metabolic diseases and microRNAs (miRNAs) are proposed as fetal programming participants. To evaluate the use of dried blood spots (DBS) on newborn screening cards (NSC) as a source of analyzable miRNAs, we optimized a commercial protocol to recover total miRNA from normal birth weight (NBW, *n* = 17–20), low birth weight (LBW, *n* = 17–20) and high birth weight (macrosomia, *n* = 17–20) newborns and analyzed the relative expression of selected miRNAs by stem-loop RT-qPCR. The possible role of miRNAs on the fetal programming of metabolic diseases was explored by bioinformatic tools. The optimized extraction of RNA resulted in a 1.2-fold enrichment of miRNAs respect to the commercial kit. miR-33b and miR-375 were overexpressed in macrosomia 9.8-fold (*p* < 0.001) and 1.7-fold, (*p* < 0.05), respectively and miR-454-3p was overexpressed in both LBW and macrosomia (19.7-fold, *p* < 0.001 and 10.8-fold, *p* < 0.001, respectively), as compared to NBW. Potential target genes for these miRNAs are associated to cyclic-guanosine monophosphate (cGMP)-dependent protein kinase (PKG), mitogen-activated protein kinase (MAPK), type 2 diabetes, transforming growth factor-β (TGF-β)and Forkhead box O protein (FoxO) pathways. In summary, we improved a protocol for analyzing miRNAs from NSC and provide the first evidence that birth weight modifies the expression of miRNAs associated to adult metabolic dysfunctions. Our work suggests archived NSC are an invaluable resource in the search for fetal programming biomarkers.

## 1. Introduction

Fetal programming or “developmental origins of adult disease” can be defined as adaptations arisen from the quality of uterine life that will determine outcomes in later life, modulating physiology and susceptibility to diseases in childhood and/or adulthood [1]. Indeed, there is evidence indicating the detrimental effects of insufficient fetal development in later outcomes, including an elevated prevalence of infectious diseases and non-communicable diseases such as obesity, type 2 diabetes (T2D) and cardiovascular diseases [2].

Fetal growth is a multifactorial process, entailing the interaction of mother, placenta and fetus with environmental factors [3]. Birth weight has long been used as an indicator of perinatal health and it is widely accepted that both low and high birth weight can have either short or long-term effects on offspring’s health. Low birth weight (LBW) neonates (<2500 g) are at elevated risk of developing dyslipidemias, hypertension and glucose intolerance [4]. On the other side, high birth weight, large for gestational age or macrosomia (>4000 g) has been associated to an increased risk of metabolic syndrome, obesity, T2D and cancer during adulthood [2].

The molecular mechanisms of fetal programming leading to disease development in adulthood are unknown but it has been proposed that epigenetic regulation by miRNAs may be playing a role since they are involved in metabolic regulation [5]. MiRNAs are short (~22 nucleotide-long), non-coding transcripts, with a complex and highly regulated biogenesis resulting in the production of single-stranded mature miRNAs acting as negative regulators of gene expression by means of a miRNA-induced silencing complex. MiRNA regulation leads to translation impairment by degradation or destabilization of target mRNAs [6]. Moreover, miRNAs contained in exosomes are released in body fluids and participate in intercellular communication, which might reflect physiological or pathological regulation of the originating cell or tissue and suggest their potential as biomarkers for clinical diagnosis [7].

Neonatal screening, a widely used routine test around the world, is applied to 90.38% of Mexican newborns during first week of life [8]. For the test, a blood sample is taken from a heel prick and stored as dried blood spots (DBS) in newborn screening cards (NSC). NSC could be a useful sampling method for nucleic acids, as they are low cost, minimally invasive, easy to transport and stable in long-term room-temperature storage. However, methodological studies on the isolation of miRNAs from NSC are so far scarce [9].

We aimed to optimize a protocol for recovering amplifiable miRNAs from DBS preserved in NSC and to analyze its expression levels in normal (NBW), low (LBW) and high birth weight (macrosomia) newborns. Afterwards, our goal was to predict probable molecular mechanisms of fetal programming for each miRNA through bioinformatics analysis of their probable target genes.

To test the utility of NSC as a source of analyzable miRNAs, we chose two miRNAs involved on insulin secretion signaling as well as obesity and T2D: miR-33b, which has been associated with glucose and lipid metabolism and insulin signaling [10,11,12] and miR-375, which has been found to be altered in induced diabetes and associated with the regulation of murine adipogenesis and insulin secretion [13,14,15]. A third miRNA, miR-454-3p, hitherto not directly related to glucose metabolism dysfunctions was also included in the study, since it is involved on regulatory inflammatory responses [16].

## 2. Results

### 2.1. Isolation of miRNAs from Dried Blood Spots on Newborn Screening Cards (NSC)

**Figure 1:**
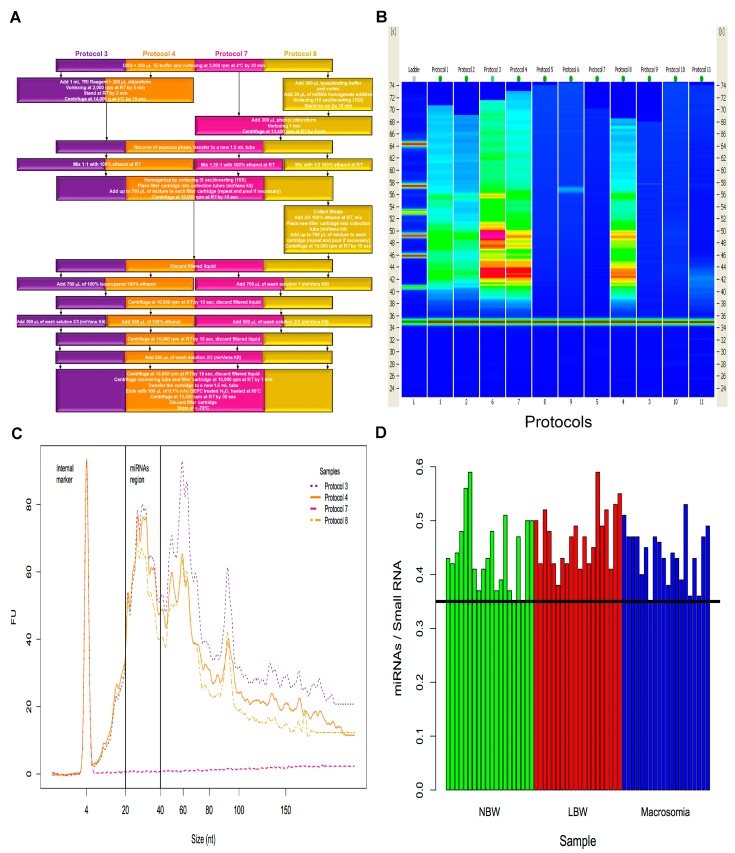
Extraction protocols and representative miRNA yield from neonatal screening cards (NSC). (**A**) Flow diagram of 4 out of 11 extraction protocols performed. (**B**) Gel-like image for small RNA quality from 11 protocols for miRNA purification performed from dried blood samples on NSC. (**C**) Electropherogram from protocols 3 (purple), 4 (orange), 7 (magenta) and 8 (gold). (**D**) miRNA:small RNA ratio from NSC of normal (green), low body weight (red) and macrosomia (blue) neonates, horizontal line indicates the 0.35 threshold ratio, each bar represents an individual, *n* = 20. NBW, normal birth weight; LBM, low birth weight.

To define the optimal conditions for miRNAs purification from dried blood spots on NSC, eleven protocols were assayed (Figure S1), based on a commercial kit (mirVana, Ambion, Austin, TX, USA) and a published protocol for dried serum spots [9]. Although five of such protocols, i.e., protocols 1–4 and 8 resulted in miRNAs concentrations above 1 ng/µL, we focused on protocols 3, 4, 7 and 8, whose extraction procedure steps are specified on Figure 1A. Bioanalyzer gel-like bands were obtained from each protocol, showing various degrees of miRNA throughput (Figure 1B). Only three of such procedures (protocols 3, 4 and 8) were enriched for miRNAs, as reflected quantitatively by electropherogram (Figure 1C). To determine miRNA:small RNA ratio, bands in the region ranging from 20 to 40 nt long were used, since this size fits the average size of miRNAs. Only samples with a ratio higher than 35% were considered reliable and were used for subsequent analysis (Figure 1D). We choose protocol 4 for miRNA’s recovery from NSC since it produced the highest yield of miRNAs at the lowest cost per sample.

### 2.2. miR-33b, miR-375 and miR-454-3p Relative Expression in Neonates with Differential Birth Weight

The expression of miR-33b, miR-375 and miR-454-3p was quantified from NSC of Mexican neonates with normal birth weight (NBW; *n* = 17–20), low birth weight (LBW; *n* = 17–20) and macrosomia (*n* = 17–20) using normalized relative quantification. All three miRNAs showed significant differences between groups (One-way ANOVA, followed by Tukey’s honest significant difference *post-hoc* test, confidence interval 95%).

**Figure 2:**
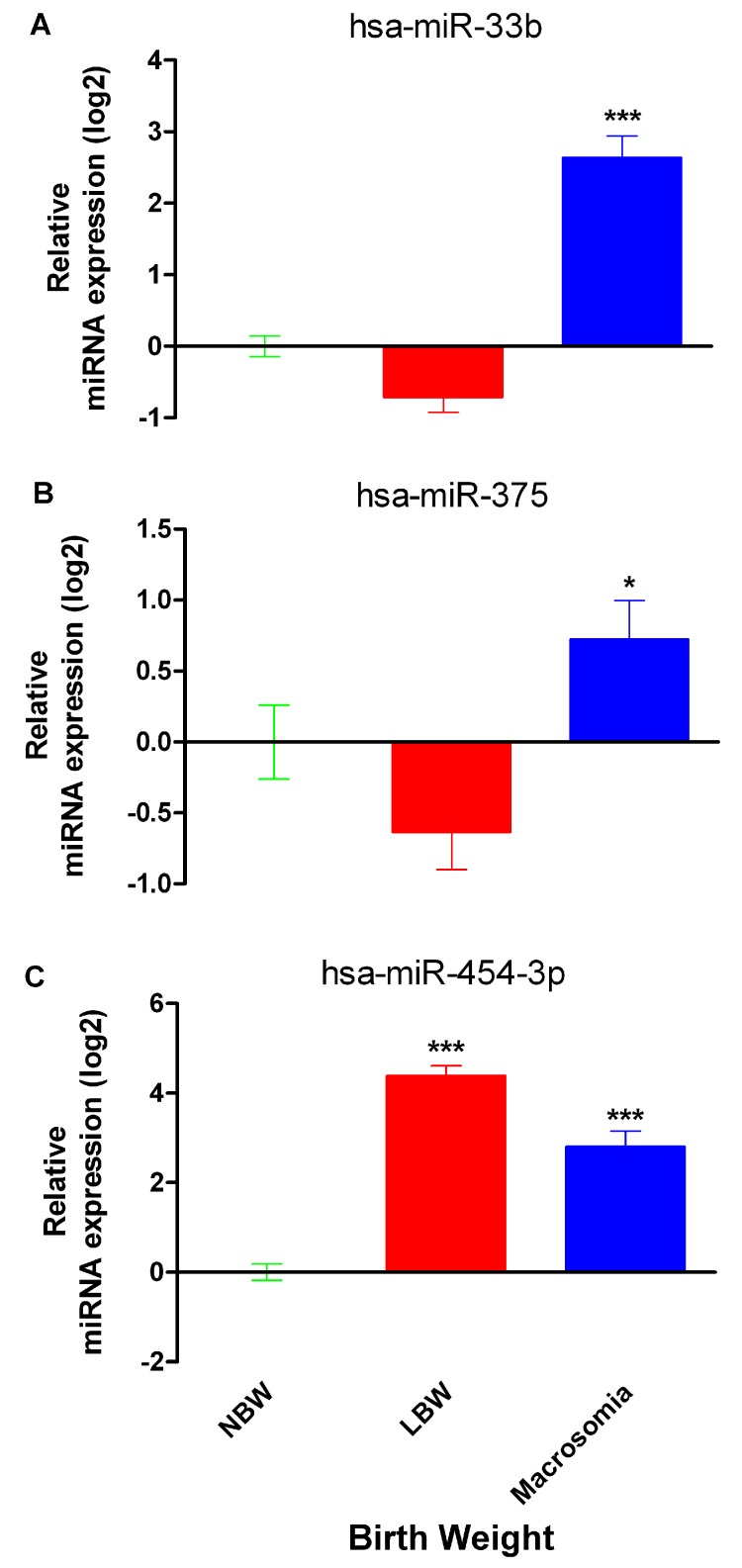
Normalized miRNA expression relative to normal birth weight. Newborn expression (log2 transformed) of miR-33b (**A**), miR-375 (**B**) and miR-454-3p (**C**) from normal birth weight (green), low birth weight (red) and macrosomia (blue) neonates. Bars represent the mean ± SEM. * (*p* < 0.05) and *** (*p* < 0.001) respect to normal birth weight, by Tukey’s honest significant difference (HSD) test. *n* = 17–20.

A significant shift was found on miR-33b expression between groups (Figure 2A), remarkably a 9.8-fold overexpression in neonates with macrosomia compared to NBW neonates (*p* < 0.001) whereas the expression of this miRNA was not significantly altered in LBW. A similar pattern was observed for miR-375 which showed a discrete increase of 1.7-fold (*p* < 0.05) in macrosomia but a not statistically significant subexpression in LBW as compared to NBW neonates (Figure 2B).

The biggest differences among groups were found for miR-454-3p relative expression. Analysis for miR-454-3p evidenced a 19.7-fold overexpression in LBW (*p* < 0.0001) and a 10.8-fold overexpression in macrosomia (*p* < 0.01) compared to NBW neonates (Figure 2C).

### 2.3. Prediction of Target Genes and Signaling Pathways for miR-33b, miR-375 and miR-454-3p

The target genes for miR-33b, miR-375 and miR-454-3p were predicted using Diana MicroT, miRanda, miRDB and PicTar algorithms. The use of multiple prediction algorithms is recommended to increase the number of candidate genes with strong likelihood to be experimentally validated [17].

**Figure 3:**
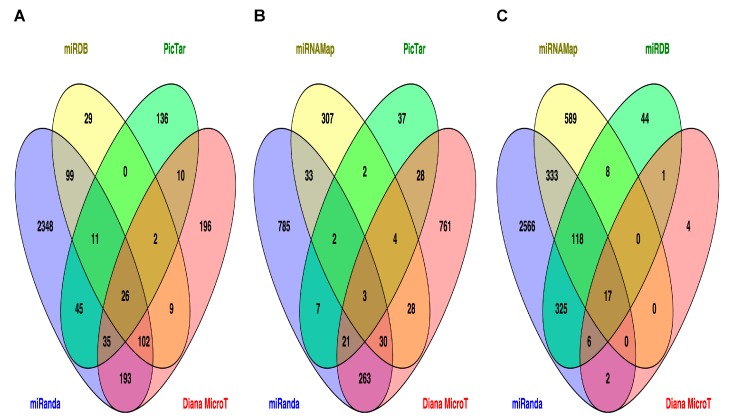
Venn diagrams illustrating the bioinformatics analysis of target genes for miR-33b (**A**), miR-375 (**B**) and miR-454-3p (**C**). For each miRNA, at least four public online database algorithms were used to identify target mRNAs. The overlap shows the number of targets shared by the algorithms, increasing the number of candidate genes with strong likelihood to be experimentally validated.

Twenty-six target genes of miR-33b were predicted by all four tools (Figure 3A). Five hundred and thirty-one target genes, predicted by at least two algorithms, were mapped to the Kyoto Encyclopedia of Genes and Genomes (KEGG) pathways using KEGG Mapper. For obtainment of enriched pathways, a Fisher’s exact test with false discovery rate (FDR) correction was calculated. As presented in Table 1, twelve possible pathways were listed with *p* < 0.05, including cyclic guanosine monophosphate (cGMP)-dependent-protein kinase (PKG) and gonadotropin releasing hormone (GnRH) signaling pathways, as well as T2D related pathway.

We also explored the possible miR-375 target genes using the bioinformatics algorithms described above (Table 2). Although we found few possible signaling pathways as possible targets for miR-375, interestingly one of these pathways involves one subtype of diabetes: maturity onset diabetes of the young.

Seventeen target genes of miR-454-3p were predicted by all tools (Figure 3C and Table S2). Eight hundred and one target genes, predicted by at least two algorithms, were mapped to KEGG pathways using KEGG Mapper. For the obtainment of enriched pathways, a Fisher’s exact test with FDR correction was calculated. As presented in Table 3, seven possible pathways were listed with a *p* < 0.05, including the signaling pathways of transforming growth factor β (TGF-β), Forkhead box O (FoxO), p53 and Hippo.

## 3. Discussion

In this study, we optimized a procedure for extracting and analyzing the expression of miRNAs present in DBS preserved on newborn screening cards (NSC). Furthermore, we present here the first evidence of a relation between birth weight and expression of circulating miRNAs in human newborns.

NSC are used for neonatal screening during the first week of life, mainly for detection of congenital metabolic disorders. Recently, these cards have also been used for single nucleotide polymorphisms analysis and even longitudinal epigenetic studies [18]. However, until now the use of NSC for the analysis of miRNAs has not been reported and methodological protocols for purification of miRNAs from adult dried blood or serum spots are scarce [9].

Our initial approach comprised 11 protocols for standardization of miRNAs extraction from neonatal DBS (Figure S1), including a solid-phase extraction with cartridges from a commercial kit (mirVana) with several modifications. We optimized a procedure for total RNA purification by replacing some of the reagents in the commercial kit for commonly used reagents (ethanol and isopropanol) and using the column-based approach for miRNA purification. Protocol number 4 resulted in a 1.2-fold enrichment of the miRNA’s fraction when compared to commercial procedure (protocol number 8, Figure 1A–D), along with a shorter processing time, an overall decrease in the cost per test and an increase of processed samples per kit. The quality and quantity of miRNA fraction obtained with our modified extraction method is suitable for analysis of miRNA expression, as showed by microfluidics electrophoresis and RT-qPCR analysis.

Several studies have aimed to improve miRNA isolation from blood fractions. Using three commercial kits, Li and Kowdley [19] showed more than 60% yield of miRNA:small RNA from serum samples. MiRNAs obtained from human plasma with a commercial kit plus carrier are also suitable for detection by microarrays [20]. Also, a modified protocol of RNAzol produced a good amplification performance of selected miRNAs from serum samples [21]. Overall, these studies address the improvement of miRNAs isolation from fresh and enough blood samples (serum and plasma). On the contrary, our technique has the potential to recover the miRNAs fraction from small, long-stored DBS samples.

To quantify the expression of miRNAs in samples obtained by our purification protocol we used stem-loop RT-qPCR [22] replacing TaqMan probes by a universal probe (UPL-21) [23]. This change allowed us to perform tests for several miRNAs with a single probe, in a less expensive approach. The simplicity of our protocol, in conjunction with the comprehensive coverage of neonatal screening worldwide, opens new possibilities to investigate the role of miRNAs in the fetal programming of adult diseases in several populations.

In our samples, the relative expression of miR-33b, a miRNA regulating the gene expression of key enzymes of fatty acid oxidation and insulin signaling components (e.g., insulin receptor substrate 2) [10]. Our results showed an increase in the expression levels of miR-33b in neonates with macrosomia, compared with NBW neonates (Figure 2A). MiR-33b and its close relative miR-33a, are located in the intronic region of genes codifying for sterol regulatory element-binding proteins (SREBPs), a family of transcription factors required for the synthesis of cholesterol and fatty acids. Interestingly, the metabolic stimulus that activates the expression of these genes also regulates the expression of both miR-33a and miR-33b and passenger strands, acting with the host gene to regulate nutrient homeostasis [11,12].

It has been reported that miR-33a and miR-33b suppress the expression of ATP-binding cassette transporter A1 (ABCA1), a crucial protein for the biogenesis of high-density lipoproteins (HDL) in the liver and are associated with the negative regulation of insulin secretion. Experimentally, it was shown that ABCA1 is a target of miR-33a and miR-145 [24]. Consequently, any alteration of cholesterol levels during early developmental stages may represent an important risk factor for cardiometabolic diseases [25]. Our in-silico analysis showed that target genes for miR-33b are also associated to cGMP-PKG and GnRH signaling pathways, as well as to insulin secretion and T2D related pathways (Table 1). Understanding the mechanisms by which miR-33b is involved in early stages of development may help to discern the etiology for lipid alterations observed in infants with macrosomia [3] and to explain its participation in the development of cardiovascular diseases and T2D.

We also analyzed the expression of miR-375 in neonates with differential birth weights since this miRNA participates in pancreatic islet development and adipogenesis in vitro, its expression could be regulated by glucose and is currently a potential target for T2D treatment [13,14,26,27]. MiR-375 was significantly overexpressed in macrosomia and non-significantly underexpressed in LBW. Previous works reported the overexpression of miR-375 in plasma of patients with T2D and the regulation of its expression by promoter methylation [27,28] and apparently by ethnic condition [28]. The overexpression of miR-375 has also been reported in pancreatic islets of leptin-deficient ob/ob mice, a model of severe insulin resistance and increased islet mass, was 30% higher than normal controls [26]. Although methylation status of miR-375 was not examined in our study, its overexpression in newborns with macrosomia suggest this miRNA is a mechanism of large for gestational age condition and comorbidities associated to this condition in adulthood. Our bioinformatics analyses suggest that target genes for miR-375 are involved in pathways related to cancer and general metabolism but also to maturity onset diabetes of the young. (Table 2).

The analysis of miR-454-3p circulating levels showed this miRNA is overexpressed in LBW and macrosomia, compared to NBW infants. There are few studies on the expression of miR-454-3p and its possible functions. One of these studies proposes miR-454-3p as a potential regulator of brain neural development, specifically in the postnatal stage [29]. In parallel, our bioinformatics analysis of potential target genes for this miRNA revealed axon guidance pathway as highly represented (Table 3).

It has also been reported an increase in the expression of miR-454-3p in neonatal umbilical cord blood monocytes in response to pro-inflammatory stimuli when compared to peripheral blood adult monocytes stimulation. This result suggests the involvement of miR-454-3p in the post-transcriptional regulation of pro-inflammatory reactions [16].

Taking into account both miR-454-3p association to neuronal development and inflammatory reactions, the results of our study could contribute to elucidate the possible molecular mechanisms explaining the propensity of LBW children to have greater burden of infectious diseases and learning problems throughout their life [2]. Also, according to the bioinformatics analysis, the target genes for miR-454-3p are involved in pathways related to cancer and general metabolism (Table 3).

Circulating miRNAs are linked to chronic degenerative diseases such as cancer or diabetes and pointed out as useful biomarkers because their specificity and presence in accessible samples (whole blood, exosomes, serum or plasma). Circulating miRNAs could be an advantageous complement to classical T2D predicting biomarkers and become targets for early intervention [30,31]. Further studies on the mechanisms by which circulating miRNAs, such as the ones explored in this study, exert their regulatory function, along with other factors such as maternal health condition, ethnic and gender of newborn, will ensure a more detailed understanding of their involvement in fetal programming.

Until now, neonatal screening has been used for early detection of metabolic dysfunctions not evident at birth and is one of the most successful public health programs [32], perhaps only surpassed by vaccination programs. The use here proposed for NSC offers a new and complementary way to harness this invaluable diagnostic resource, which will allow us to deepen in the mechanisms of fetal programming and its transgenerational inheritance [33,34].

## 4. Materials and Methods

### 4.1. Ethics Statement

This study was approved by the Institutional Ethical Committee of the Instituto Potosino de Investigación Científica y Tecnológica in accordance with the Declaration of Helsinki of 1975 as revised in 2013 (FOMIXSLP-195024/30 October 2013). Newborn screening cards (NSC) were donated by the Laboratorio Estatal de Salud Pública of San Luis Potosí, México. Parents informed consent was not required since Mexican regulation states property of NSC belongs to health authorities. Data were analyzed anonymously and researchers had no knowledge of other clinical or identity information, except for the one declared on this manuscript.

Neonatal birth weights were defined as follows: low birth weight (LBW): ≤2500 g, normal birth weight (NBW): 2501–4299 g and high birth weight or macrosomia: ≥4300 g, according to World Health Organization percentile criteria [35]. Each group consisted of 20 samples from full term mixed gender neonates (37–41 weeks of gestation), born during 2013–2014.

### 4.2. RNA Purification

NSC stored at room temperature (~22 °C) for up to 2 years in plastic sterile bags with desiccant and DBS of at least 8 mm diameter were used to assure blood volumes of 20–50 µL per sample and to avoid the risk of false negatives as reported for metabolic analyses [36], although use of blood spots larger than 1 cm did not change the miRNA recovery yield. Total RNA was isolated from NSC, using 11 different protocols (Table S1), which are modified versions of mirVana kit (Life Technologies, Carlsbad, CA, USA) and Patnaik et al., 2010 [9] protocols. Briefly, 300 µL of TE buffer (Tris-HCl 10 mM, EDTA 1 mM, pH 7.6) were added to a 1.5 mL tube containing an individual 1 cm diameter circle of dried blood sample in NSC for rehydration and vortexed at 2000 rpm at 4 °C for 30 min using a multi-tube holder on a Genie 2 vortex (Fisher Scientific, Waltham, MA, USA). The process was continued using either a protocol for small RNAs enrichment or for total RNA (Table S1), adding differentially to each protocol either TRI Reagent (Sigma-Aldrich, St. Louis, MO, USA) and chloroform, or a mix of phenol-chloroform or following manufacturer’s instructions for the mirVana kit. The different procedures followed kept the use of filter cartridges and collection tubes (mirVana kit) but different washing solutions were assayed. Finally, RNA was eluted with 100 µL of DEPC 0.1% (*v*/*v*) treated water at 95 °C and centrifuged at 13,400 rpm for 30 s. All samples were analyzed in the 2100 Bioanalyzer (Agilent Technologies, Santa Clara, CA, USA) using the Small RNA Assay Kit (Agilent Technologies, Santa Clara, CA, USA) following the Agilent Small RNA kit guide. Samples were kept at ≤−70 °C for long-term storage.

### 4.3. cDNA Synthesis

Total RNA of <2 months of purification, was subjected to retrotranscription using a specific stem-loop RT primer as described by Chen et al [22]. All mature miRNAs sequences were obtained from miRBase v20 [37]. Primers were designed using miRNA primer design tool Software [23]. Primer sequences are specified in Table 4 and were synthesized by Integrated DNA Technology (IDT, Coralville, IA, USA). All primers were analyzed for secondary structure using OligoAnalyzer 3.1 software (http://www.idtdna.com/analyzer/Applications/OligoAnalyzer/).

Stem-loop pulsed reverse transcription reaction was carried out [38]. Briefly, a fixed volume of RNA was used for all samples (2 µL), plus 1 µL of stem-loop primer (100 µM), 2 µL of dNTP mix (10 mM mix), 0.1 µL of M-MLV reverse transcriptase (200 U/µL) (Promega, Madison, WI, USA), 4 µL of M-MLV RT 5× Reaction Buffer, 0.032 µL of RNasin inhibitor (2500 U/µL) and nuclease free water was added to a final volume of 20 µL per reaction. RT reactions were incubated at 16 °C for 30 min, followed by retrotranscription for 60 cycles at 30 °C for 30 s, 42 °C for 30 s and 50 °C for 1 s and terminated by incubating at 85 °C for 5 min [38]. All reactions included a minus reverse transcriptase enzyme reaction as negative control, since RNA preparations were not treated with DNase. Samples were stored at −20 °C until use. cDNA was diluted 1:5 before use.

### 4.4. Real Time Quantitative PCR

Sequences of specific forward primers are listed on Table 4, a universal reverse primer (URP) and universal probe library probe #21 (UPL-21) were the same for all miRNAs, according to Czimmerer et al. method [23]. qPCR reactions were carried out on a Roche’s LightCycler 2.0 (Roche Diagnostics, Mannheim, Germany) using QuantiTect Probe PCR Kit (Qiagen, Hilden, Germany) in a final volume of 20 µL containing: 5 µL of cDNA dilution, 0.1 µL of 100 µM specific reverse primer, 0.1 µL of 100 µM URP, 0.2 µL of probe UPL #21 (Roche) and 4.6 µL of nuclease free water; PCR reactions were performed by duplicate. PCR conditions were: initial denaturation at 95 °C for 10 min, followed by 50 cycles of 95 °C for 10 s, 60 °C for 30 s and 72 °C for 1 s, with a final hold at 40 °C for 10 min. There was no detection signal in non-template control (NTC) or omitting reverse transcriptase enzyme reaction (negative control) after 50 cycles of amplification.

Since there are none normalizing genes for circulating miRNAs analysis in human neonates, to determine the endogenous reference genes for normalization by relative quantification method, we assessed adult circulating miRNAs reported in the literature as suitable reference genes: miR-24 [39], miR-106a-5p [40] and miR-16-5p [41]. NormFinder algorithm was used [42] to identify the stability value of internal candidate reference genes and the best combination of two genes with the lowest quantification cycle (Cq) coefficient of variation. Stability value for miR-106a-5p and miR-16-5p was 0.011 and 0.007, respectively.

Hence, the relative abundance of selected miRNAs (33b, 375 and 454-3p) from NSC from neonates with different body weight was normalized to miR-106a-5p and miR-16-5p as reference miRNAs. Relative quantification was obtained using the double delta threshold cycle ($2^{-\Delta \Delta Ct}$) method [43] and the geometric mean of miR-106a-5p and miR-16-5p [44] relative to samples of NBW [43], followed by base 2 logarithm (log2) transformation.

### 4.5. Metabolic Pathway Analysis

Target genes for miR-33b, miR-375 and miR-454-3p were predicted using at least four of these public database algorithms online: Diana MicroT, miRanda, miRDB, PicTar and miRNAMap. Target genes predicted by at least two different tools, were mapped to Kyoto Encyclopedia of Genes and Genomes (KEGG) pathways using KEGG Mapper [45] and enriched by a Fisher’s exact test (confidence interval 95%) with FDR correction using R Software (R Development Core Team, 2013).

### 4.6. Statistical Analysis

To test whether the data of each group of newborns (normal, low and high birth weights), presented differences on miRNAs’ relative expression, one-way ANOVA was performed followed by Tukey’s HSD *post-hoc* test (confidence interval 95%). Statistical analyses were done using R Software and GraphPad Prism version 5.00 (GraphPad Software Inc., San Diego, CA, USA).

## 5. Conclusions

The present study stands a precedent on the usefulness of employing NSC for recovering analyzable miRNAs which might be participating on the fetal programming of adult diseases. Our work also provides the first evidence that birth weight, an early predictor for metabolic diseases, modifies the expression of miRNAs associated to adult metabolic dysfunctions. Thus, our work could be useful in the design of further studies on the role of miRNAs as fetal programming biomarkers which also should include additional clinical data on fetal and maternal conditions [31].

## Figures and Tables

**Table 1:** Target genes of miR-33b by Kyoto Encyclopedia of Genes and Genomas (KEGG) pathway analysis.

KEGG Pathway	*p* Value	Faslse Discovery Rate Adjustment	KEGG ID
Axon guidance	0.0001975	0.00237	ko04360
cGMP-PKG signaling pathway	0.01559	0.00237	ko04022
Type 2 diabetes mellitus	0.01987	0.00237	ko04930
Adherens junction	0.02716	0.00237	ko04520
GnRH signaling pathway	0.02716	0.04484	ko04912
Glutamatergic synapse	0.02894	0.04484	ko04724
Pathogenic Escherichia coli infection	0.02926	0.04484	ko05130
Cholinergic synapse	0.03329	0.04484	ko04725
Amphetamine addiction	0.03363	0.04484	ko05031
Insulin secretion	0.03774	0.04484	ko04911
Nicotine addiction	0.0423	0.04501	ko05033
Vascular smooth muscle contraction	0.04501	0.04501	ko04270

**Table 2:** Target genes of miR-375 by KEGG pathway analysis.

KEGG Pathway	*p* Value	FDR Adjustment	KEGG ID
Glutamatergic synapse	0.0108	0.03308333	ko04724
Transcriptional misregulation in cancer	0.01638	0.03308333	ko05202
Maturity onset diabetes of the young	0.01985	0.03308333	ko04950
Mitogen-activated protein kinase (MAPK) signaling pathway	0.03948	0.03979	ko04010
Axon guidance	0.03979	0.03979	ko04360

**Table 3:** Target genes of miR-454-3p by KEGG pathway analysis.

KEGG Pathway	*p* Value	FDR Adjustment	KEGG ID
Endocytosis	0.001724	0.012068	ko04144
TGF-β signaling pathway	0.01607	0.04421	ko04350
Axon guidance	0.02799	0.04421	ko04360
FoxO signaling pathway	0.03921	0.04421	ko04068
p53 signaling pathway	0.03971	0.04421	ko04115
Proteoglycans in cancer	0.04393	0.04421	ko05205
Hippo signaling pathway	0.04421	0.04421	ko04390

**Table 4:** Primer sequences used for RT-qPCR.

Name	Sequence (5′–3′)	Accession Number of Mature miRNA
hsa-miR-33b RT stem-loop	GTTGGCTCTGGTGCAGGGTCCGAGGTATTCGCACCAGAGCCAACGCAATG	MIMAT0003301
hsa-miR-33b specific forward	GTTTGGGTGCATTGCTGTTG	
hsa-miR-375 RT stem-loop	GTTGGCTCTGGTGCAGGGTCCGAGGTATTCGCACCAGAGCCAACTCACGC	MIMAT0000728
hsa-miR-375 specific forward	TGGTTTTTGTTCGTTCGGCT	
hsa-miR-454-3p RT stem-loop	GTTGGCTCTGGTGCAGGGTCCGAGGTATTCGCACCAGAGCCAACACCCTA	MIMAT0003885
hsa-miR-454-3p specific forward	GGTGTGGTAGTGCAATATTGCTTA	
hsa-miR-106a RT stem-loop	GTTGGCTCTGGTGCAGGGTCCGAGGTATTCGCACCAGAGCCAACCTACCT	MIMAT0000103
hsa-miR-106a specific forward	TGGGTAAAAGTCCTTACAGTGC	
hsa-miR-16-5p RT stem-loop	GTTGGCTCTGGTGCAGGGTCCGAGGTATTCGCACCAGAGCCAACCGCCAA	MIMAT0000069
hsa-miR-16-5p specific forward	TGTTTTTTTTTGTAGCAGCACGTAAATA	
Universal reverse primer	GTGCAGGGTCCGAGGT	NA
Universal ProbeLibrary probe #21	TGGCTCTG	NA

## Supplementary Materials

Supplementary materials can be found at www.mdpi.com/1422-0067/18/12/2552/s1.

## Abbreviations

DBS	dried blood spots
NSC	newborn screening cards
LBW	low birth weight
NBW	normal birth weight
T2D	type 2 diabetes mellitus
