# Supplementary material for: Analysis of MicroRNA Expression in Newborns with Differential Birth Weight Using Newborn Screening Cards
Source: Int J Mol Sci. 2017 Nov 28;18(12):2552. doi: 10.3390/ijms18122552 (PMC5751155; doi:10.3390/ijms18122552)
Supplement: Supplementary file 1 [file ijms-18-02552-s001.zip › Supplementary Captions.docx]

**Supplementary Materials**

**S1 Fig.** Representative miRNA concentration from extraction protocols. Comparative miRNA yield of the 11 extraction protocols of DBS. Horizontal blue line separates the protocols that resulted in miRNAs concentrations above 1 ng/µl.

**S1 Table.** Description of standardization for miRNAs extraction protocols.

**S2 Table.** Bioinformatics analysis of potential target genes for miRNAs 33b, 375 and 454-3p.
