# Supplementary material for: Analysis of MicroRNA Expression in Newborns with Differential Birth Weight Using Newborn Screening Cards
Source: Int J Mol Sci. 2017 Nov 28;18(12):2552. doi: 10.3390/ijms18122552 (PMC5751155; doi:10.3390/ijms18122552)
Supplement: Supplementary file 1 [file ijms-18-02552-s001.zip › S1 Table.pdf]

S1 Table. Description of Standardization for miRNAs Extraction Protocols.

<table>
<tr><th colspan="11">Number of Protocol</th></tr>
<tr><th>1</th><th>2</th><th>3</th><th>4</th><th>5</th><th>6</th><th>7</th><th>8</th><th>9</th><th>10</th><th>11</th></tr>
<tr><td colspan="11">DBS + 300 µl TE buffer and vortexing at 2,000 rpm at 4°C by 30 min</td></tr>
<tr><td colspan="4">Add 1 ml TRI Reagent + 200 µl chloroform</td><td colspan="2">Add 1.2 ml phenol:chloroform</td><td rowspan="2"></td><td>Add 300 µl lysis/binding buffer and vortex vigorously
Add 30 µl of miRNA homogenate additive</td><td>Add 1 ml TRI Reagent + 200 µl chloro-form</td><td>Add 1.2 ml phenol: chloroform</td><td>Add 1 ml TRI Reagent + 200 µl chloroform</td></tr>
<tr><td colspan="6" rowspan="3">Vortexing at 2,000 rpm at RT by 5 min
Stand at RT by 2 min
Centrifuge at 14,000 g at 4°C by 15 min</td><td>Vortexing (10 sec)/inverting (10X)
Stand on ice by 10 min</td><td colspan="3" rowspan="3">Vortexing at 2,000 rpm at RT by 5 min
Stand at RT by 2 min
Centrifuge at 14,000 g at 4°C by 15 min</td></tr>
<tr><td colspan="2">Add 300 µl phenol:chloroform
Vortexing 1 min</td></tr>
<tr><td colspan="2">Centrifuge at 13,400 rpm at RT by 5 min</td></tr>
<tr><td colspan="11">Recover of aqueous phase, transfer to a new 1.5 ml tube</td></tr>
<tr><td colspan="6">Mix 1:1 with 100% ethanol at RT</td><td>Mix 1.25:1 with 100% ethanol at RT</td><td colspan="3">Mix with 1/3 100% ethanol at RT</td><td>Mix 1:1 with 100% ethanol at RT</td></tr>
<tr><td colspan="11">Homogenize by vortexing (5 sec)/inverting (10X)
Place filter cartridge into collection tubes (mirVana kit)
Add up to 700 µl of mixture to each filter cartridge (repeat and pool if necessary)
Centrifuge at 10,000 rpm at RT by 15 sec</td></tr>
</table>

<table>
<tr><td colspan="5"></td><td>Collect filtrate
Mix with 2/3 100% ethanol at RT
Mix vigorously
Place new filter cartridge into collection tube (mirVana kit)
Add up to 700 µl of mixture to each cartridge (repeat and pool if necessary)
Centrifuge at 10,000 rpm at RT by 15 sec</td></tr>
<tr><td colspan="6">Discard filtered liquid</td></tr>
<tr><td>Add 700 µl of 100% isopropa-nol: 100% ethanol</td><td>Add 700 µl of wash solution 1 (mirVa-na Kit)</td><td colspan="2">Add 700 µl of 100% isopropanol:100% ethanol</td><td colspan="2">Add 700 µl of wash solution 1 (mirVana Kit)</td></tr>
<tr><td colspan="6">Centrifuge at 10,000 rpm at RT by 15 sec, discard filtered liquid</td></tr>
<tr><td>Add 500 µl of 100% ethanol</td><td colspan="2">Add 500 µl of wash solution 2/3 (mirVana Kit)</td><td>Add 500 µl of 100% ethanol</td><td colspan="2">Add 500 µl of wash solution 2/3 (mirVana Kit)</td></tr>
<tr><td colspan="6">Centrifuge at 10,000 rpm at RT by 15 sec, discard filtered liquid</td></tr>
<tr><td>Add 500 µl of 100% ethanol</td><td colspan="5">Add 500 µl of wash solution 2/3 (mirVana Kit)</td></tr>
<tr><td colspan="6">Centrifuge at 10,000 rpm at RT by 15 sec, discard filtered liquid
Centrifuge recovering tube and filter cartridge at 10,000 rpm at RT by 1 min
Transfer the cartridge to a new 1.5 ml tube
Elute with 100 µl of 0.1% (v/v) DEPC treated H_2O, heated at 95°C
Centrifuge at 13,400 rpm at RT by 30 sec
Discard filter cartridge
Store at < -70°C</td></tr>
</table>

In Protocol number 8, mirVana kit's (Life Technologies) reagents were used for purification and enrichment for small RNAs.
