# Supplementary material for: Analysis of MicroRNA Expression in Newborns with Differential Birth Weight Using Newborn Screening Cards
Source: Int J Mol Sci. 2017 Nov 28;18(12):2552. doi: 10.3390/ijms18122552 (PMC5751155; doi:10.3390/ijms18122552)
Supplement: Supplementary file 1 [file ijms-18-02552-s001.zip › S2 Table.pdf]

S2 Table. Bioinformatics analysis of potential target genes for miRNAs 33b, 375 and 454-3p.

List of potential target genes for miR-33b:

Gene ID	Gene name	Location
ABCA1	ATP-binding cassette, sub-family A (ABC1), member 1	9q31.1
YWHAH	Tyrosine 3-monooxygenase/tryptophan 5-monooxygenase activation protein	22q12.3
MAP3K7	Mitogen-activated protein kinase kinase kinase 7	6q15
B3GALT2	UDP-Gal:betaGlcNAc beta 1,3-galactosyltransferase, polypeptide 2	1q31
SLC25A25	Solute carrier family 25 (mitochondrial carrier; phosphate carrier), member 25	9q34.11
ATP1B1	ATPase, Na+/K+ transporting, beta 1 polypeptide	1q24
SATB2	SATB homeobox 2	2q33
ZNF281	Zinc finger protein 281	1q32.1
CLPX	Caseinolytic mitochondrial matrix peptidase chaperone subunit	15q22.31
EN2	Engrailed homeobox 2	7q36
SLC26A7	Solute carrier family 26 (anion exchanger), member 7	8q23
GRIA3	Glutamate receptor, ionotropic, AMPA 3	Xq25
NAA15	N(alpha)-acetyltransferase 15, NatA auxiliary subunit	4q31.1
SHANK2	SH3 and multiple ankyrin repeat domains 2	11q13.2
RAP2A	RAP2A, member of RAS oncogene family	13q34
GDI2	GDP dissociation inhibitor 2	10p15

Gene ID	Gene name	Location
EPHA8	EPH receptor A8	1p36.12
KPNA4	Karyopherin alpha 4 (importin alpha 3)	3q25.33
PDGFRA	Platelet-derived growth factor receptor, alpha polypeptide	4q12
ST18	Suppression of tumorigenicity 18, zinc finger	8q11.23
MLLT3	Myeloid/lymphoid or mixed-lineage leukemia	9p22
CDK5R1	Cyclin-dependent kinase 5, regulatory subunit 1 (p35)	17q11.2
HADHB	Hydroxyacyl-CoA dehydrogenase/3-ketoacyl-CoA thiolase/enoyl-CoA hydratase (trifunctional protein), beta subunit	2p23
MORF4L1	Mortality factor 4 like 1	15q24
BCL11A	B-cell CLL/lymphoma 11A (zinc finger protein)	2p16.1
PRICKLE2	Prickle homolog 2 (Drosophila)	3p14.1

List of potential target genes for miR-375:

Gene ID	Gene name	Location
RLF	Rearranged L-myc fusion	1p32
HOXD3	Homeobox D3	2q31.1
CTGF	Connective tissue growth factor	6q23.1

List of potential target genes for miR-454-3p:

Gene ID	Gene name	Location
SMARCD2	SWI/SNF related, matrix associated, actin dependent regulator of chromatin, subfamily d, member 2	17q23.3
TSHZ1	Teashirt zinc finger homeobox 1	18q22.3
WHSC1L1	Wolf-Hirschhorn syndrome candidate 1-like 1	8p11.2
RAB5A	RAB5A, member RAS oncogene family	3p24-p22
PAN3	PAN3 poly(A) specific ribonuclease subunit	13q12.2
HIVEP2	Human immunodeficiency virus type I enhancer binding protein 2	6q23-q24
FRZB	Frizzled-related protein	2q32.1
STIM2	Stromal interaction molecule 2	4p15.2
MET	MET proto-oncogene, receptor tyrosine kinase	7q31
SLAIN1	SLAIN motif family, member 1	13q22.3
MIER1	Mesoderm induction early response 1, transcriptional regulator	1p31.3
LRP12	Low density lipoprotein receptor-related protein 12	8q22.2
EIF2C4	Argonaute RISC catalytic component 4	1p34
PTPRG	Protein tyrosine phosphatase, receptor type, G	3p21-p14
PHF20	PHD finger protein 20	20q11.22-q11.23
JARID2	Jumonji, AT rich interactive domain 2	6p24-p23
LCOR	Ligand dependent nuclear receptor corepressor	10q24
