# Supplementary figures and images for: Analysis of MicroRNA Expression in Newborns with Differential Birth Weight Using Newborn Screening Cards
Source: Int J Mol Sci. 2017 Nov 28;18(12):2552. doi: 10.3390/ijms18122552 (PMC5751155; doi:10.3390/ijms18122552)

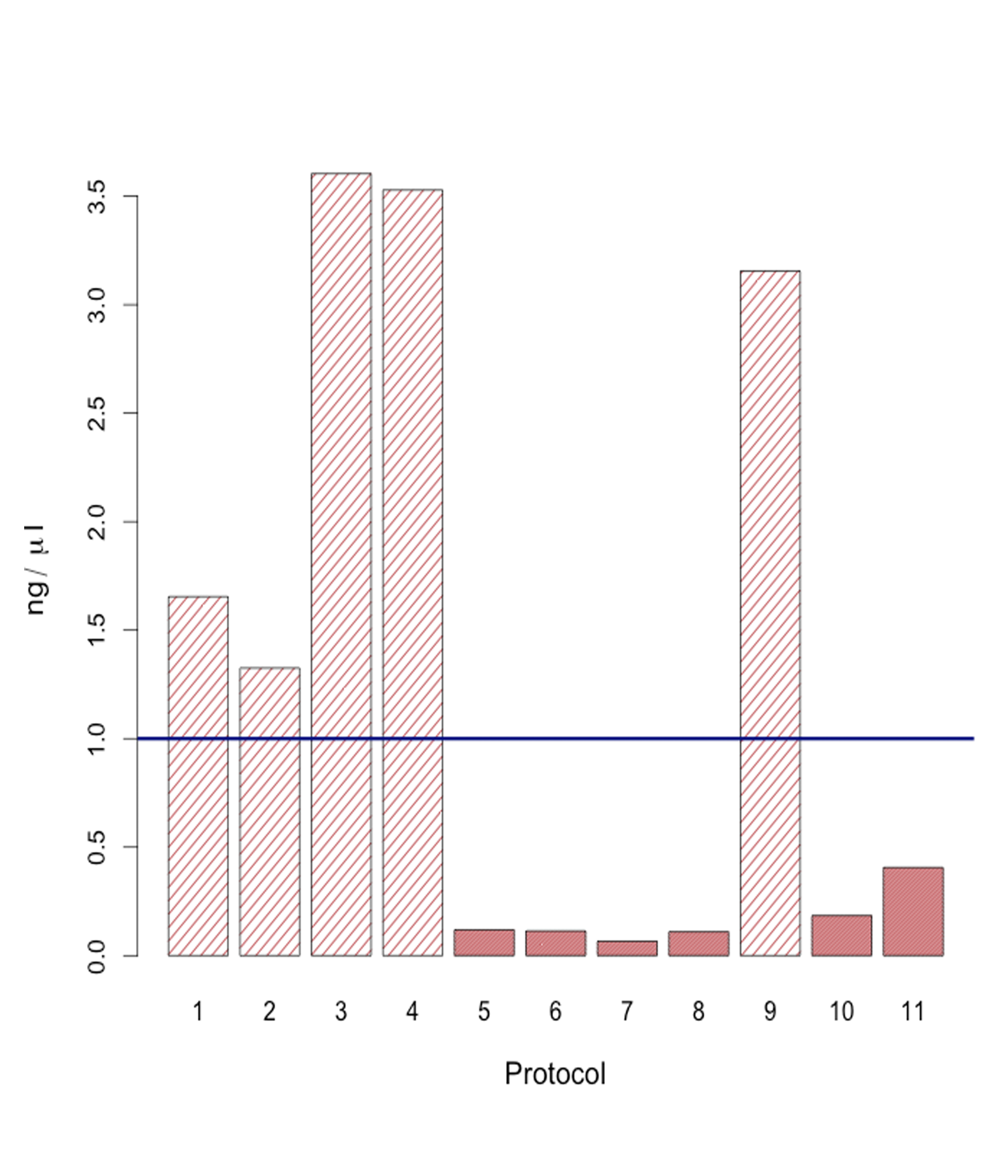

Supplement: Supplementary file 1 [file ijms-18-02552-s001.zip › S1 Figure.tif]
